# What Do We Know about Opioids and the Kidney?

**DOI:** 10.3390/ijms18010223

**Published:** 2017-01-22

**Authors:** Mary Mallappallil, Jacob Sabu, Eli A. Friedman, Moro Salifu

**Affiliations:** Department of Internal Medicine, State University of New York at Downstate, Brooklyn, New York, NY 11203, USA; sabujacob111@gmail.com (J.S.); elifriedmn@aol.com (E.A.F.); moro.salifu@downstate.edu (M.S.)

**Keywords:** opioids, renal failure, pharmacokinetics

## Abstract

Evidence suggests a link between opioid use and kidney disease. This review summarizes the known renal manifestations of opioid use including its role in acute and chronic kidney injury. Both the direct and indirect effects of the drug, and the context which leads to the development of renal failure, are explored. While commonly used safely for pain control and anesthesia in those with kidney disease, the concerns with respect to side effects and toxicity of opioids are addressed. This is especially relevant with the worldwide increase in the use of opioids for medical and recreational use.

## 1. Introduction

The universal increase in opioid use requires practitioners to be comfortable with the use of these medications, especially in those with organ dysfunction such as kidney disease to avoid complications. This article targets to highlight the safe use of opioids in those with kidney disease. Pain occurs when a signal from a noxious stimulus is transmitted via peripheral nerve fibers to the spino-thalamic tract, and finally relayed to the somatosensory cortex. Opioid receptors are present in the central and peripheral nervous systems, and when they are activated, further transmission of the signal to higher pain centers are blocked. Opioid analgesics work by binding to these receptors, resulting in a decreased perception of pain through second messenger G proteins pathways. Due to the receptors in peripheral tissue, they can also be used for cough, intractable diarrhea and for dyspnea. However, they may also cause side effects like euphoria, generalized central nervous system (CNS) depression, respiratory depression, itching, constipation, urinary retention, and in the elderly population, an increased risk of falls. There are three types of opioid receptors (delta, mu and kappa) and all clinically useful opioids are mu receptor agonists. (See Table 1 for commonly prescribed opioids and relative strengths).

## 2. Why Has Opioid Use Increased?

Due to the euphoric effect of opioids, they are targets of abuse with troublesome consequences. Over the past decade there has been an increase in mortality, both in the United States and worldwide from opioid overdose. Over this period, prescription opioid abuse has reached alarming proportions in the United States [1,2]. A systematic review of 169 studies conducted between 1980 and 2013, found varying prevalence in mortality rates from opioid overdose; those that reported long-term trends revealed increases over time of overdose-related deaths or hospitalizations [3]. Recent data from the National Institute on Drug Abuse noted that the prevalence of heroin abuse and dependence in young adults has increased significantly [4]. In New York City, the mortality rate from drug overdoses tripled between 1990 and 2006 [5]; it is not limited to being an inner city problem and widespread use of prescription drugs for non-medical use was noted among rural youth in the USA [6]. In adults, approximately 5% reported nonmedical opioid use; about twelve million Americans have used opioids for nonmedical purposes and among these, many have been abusing more than one narcotic agent [7]. Repeated opiate and cocaine use (more than 5 times in a lifetime) was noted to be associated with increased prevalence in reduced kidney function [8]. In addition, prescription opioid use was noted to be associated with higher albuminuria when compared to non-opioid users [9].

In terms of medical use in the elderly without a cancer diagnosis, United States national Medicare data between 2007 and 2012 revealed a correlation between opioid prescriptions for more than 90 days with toxicity, and an increase in the number of prescriptions in this group from 4.6% in 2007 to 7.35% in 2012. Age and female gender were individual factors that were noted to be more likely for long-term opioid use and this increased the opioid related emergency room visit odds by 60% [10]. The population of the elderly and those with chronic kidney disease (CKD) is rising globally [11,12]. Elderly patients suffer from more pain-causing conditions, and have more adverse drug effects related to polypharmacy. This was noted in the CONCERT study, which explored opioid use for non-cancer pain in 4 million people between 1997 and 2005, and showed an increase of use in women, in those with advanced age, and a doubling of the opioid use between 1997 and 2005 [13].

One of the reasons for the rise in use of opioids in the aging population is the ceiling effect (see Table 2). In pharmacology, the ceiling effect for a drug is reached when further increments of the drug has diminishing desired effects and may start to have unwanted side effects. Opioids are begun at starting doses, and increased to reach the effective dose. Pure agonists have a wide therapeutic dosing range without an absolute ceiling effect—there will continue to be benefits with dose increases, so there is no real upper limit for the medication—this is particularly true of morphine. Incremental dosing needs to be balanced with side effects of respiratory depression, constipation and depressed sensorium. Mixed agonist-antagonist opioids have a ceiling effect and are limited in the pain control they can achieve. When compared to non-opioid pain medications like non-steroidal anti-inflammatory agents, tricyclic antidepressants, and anticonvulsants, opioids are easier to use. Non-opioids have a narrow therapeutic dose range in which organ damage can still occur, and have a strong ceiling effect, resulting in greater prescriber comfort of opioids in the elderly [14].

## 3. Context of Renal Failure from Opioid Use

It is important to note that opioids are commonly used safely in anesthesia and pain control in the perioperative period in those with kidney disease. The renal toxicity appears in the context of inappropriate use: either inadvertently higher than needed doses, in the presence of other toxins, with pre-existing dehydration, or prostate enlargement. Chronic use of opioids, as noted by Novick et al. [10], results in greater incidence of toxicity due to accumulation of metabolites, which could cause unwanted side effects. One reason is that with chronic use, a steady state of the drug is reached with distribution and accumulation in the various body compartments. With a pro-drug that is metabolized to morphine, both the drug and the intermediary metabolite levels may also build up in the various compartments, resulting in unwanted side effects.

Opioid overdose can result in acute kidney injury (AKI) due to dehydration, hypotension, rhabdomyolysis, and urinary retention. CKD may result due to the mode of administration of the drug: skin-popping resulting in amyloidosis. Heroin-associated nephropathy (HAN) is now considered to be related to a toxin introduced into the heroin during the processing of the drug. Administration methods like intravenous drug abuse may also result in the spread of hepatitis B and C and human immunodeficiency virus (HIV). There have been three case reports of renal lipoidosis on kidney biopsy in patients on methodone, however, confounding factors, including hepatitis B and C, were noted in these cases [15].

In the elderly, important age-related changes might alter opioid drug pharmacokinetics and result in unwanted side effects. Decreased organ function of the liver and kidney, in addition to alterations in adipose tissue composition, occur, altering opioid pharmacokinetics that allow metabolites to accumulate and be present for longer [16]. Most opioids undergo oxidation, (exceptions being morphine and buprenorphine). Metabolites may undergo hydroxylation in the intestine, then enter hepatic circulation prior to finally being excreted either in the feces or urine. In the elderly, hepatic blood flow decreases and age-related changes that occur in cytochromes, with conjugation reactions in the body, can increase the risk of toxicity and drug-drug interactions [17]. Both liver and kidney function alterations that occur with age result in a higher risk of toxicity of opioids alone or in combination with other drugs [18].

In the context of CKD, an increasingly important finding in nephro-pharmacology has been the unexpected alterations in the non-renal metabolism of drugs that had led to changes in Food & Drug Administration’s guidance to industry [19].

## 4. Incidence of Renal Failure from Opioid Use

The true incidence of renal failure from opioid use is not well defined in part due to under recognition and under reporting. The usual mechanism for AKI with opioid use is in the setting of multi-organ failure from respiratory depression, hypoxia, and volume depletion with or without rhabdomyolysis [20]. Rhabdomyolysis may be seen commonly with AKI due to many toxins including alcohol, heroin, cocaine and synthetic cannabinoids [21]. Multiple toxins may be involved in causing rhabdomyolysis. It was noted that exogenous toxins were the most common cause of rhabdomyolysis, with illicit drugs, alcohol, and prescribed drugs responsible for 46% of rhabdomyolysis in hospitalized patients. There is no data captured on those that were not hospitalized. In 60% of all cases, multiple factors were present. In those with rhabdomyolysis, AKI was diagnosed in 46% of patients and resulted in 3.4% mortality. Rhabdomyolysis is a common cause of AKI, leading to 9% of all cases of AKI in the USA [22]. Rhabdomyolysis may be seen with heroin overdose and from opioid withdrawal, resulting in muscle injury as well [23]. The incidence of AKI from urinary retention due to opioids, while undefined, would be greater in the elderly, especially if there was the presence of other risk factors like benign prostatic hypertrophy or medications like anti-cholinergic agents.

## 5. Mechanisms of Kidney Damage with Opioid Use

**Figure 1:**
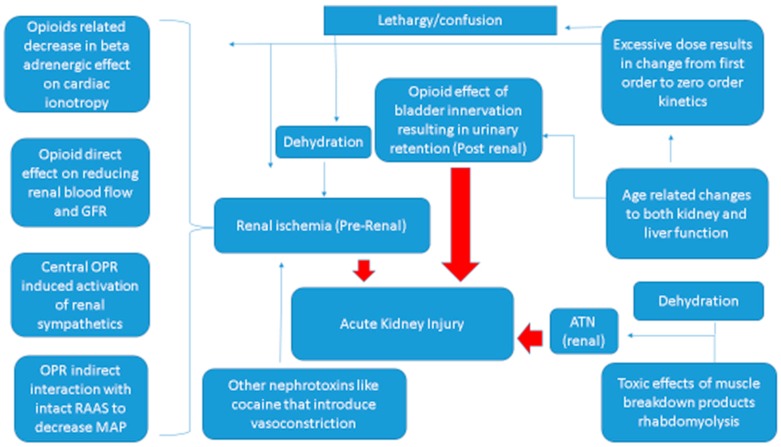
Mechanism of opioid-related kidney failure: pre-renal, acute tubular necrosis and post-renal failure. GFR: glomerular filtration rate; MAP: mean arterial pressure; OPR: opioid peptide receptors; RAAS: renin–angiotensin–aldosterone system; ATN: acute tubular necrosis. Red arrows show the final pathway to acute kidney injury.

There are complex interactions within the body’s neuroendocrine systems in response to opioids that are described below, including alterations in the autonomic nervous system (sympathetic and parasympathetic nervous system), the renin–angiotensin–aldosterone system and anti-diuretic hormone. In addition, there are also other mechanisms involved including dehydration, rhabdomyolysis and urinary retention. These changes may be measured by levels of neurotransmitters, hormones, cytokines and various peptides. The levels of the factors being measured are specific to the site where they are measured (cardiac, plasma) and their role and as either initiating or as a compensatory response. Further local versus systemic levels of the substance and additive pathologic states due to co-morbidities also need to be considered in the pathophysiology of opioid related kidney damage (See Figure 1).

### 5.1. Enhancing Para-Sympathetics and Reducing Sympathetic Effect

Changes in the sympathetic and parasympathetic nervous systems affect the kidney by altering renal blood flow and glomerular filtration rate. These changes occur at several levels including the heart and kidney. Usually, the autonomic nervous system controls vital body functions with the sympathetic and parasympathetic innervation acting antagonistically based on need. In the cardiovascular system, the sympathetic nervous system (SNS) increases heart rate and myocardial contractility, and raises peripheral vascular resistance and arterial blood pressure (BP) via vasoconstriction. In the myocardium, sympathetic β-adrenergic receptors (β-AR) increase the heart rate and contractility; this is opposed by the parasympathetic effect mediated by the vagus nerve. In studies, vagus nerve activity is enhanced by opioids resulting in bradycardia and a decrease in cardiac ionotropy [24,25].

Opioid peptide receptors (OPR) are activated by endorphins (see Table 1), of which 20 or so have been identified to date. OPR activation can inhibit cardiac excitation-contraction coupling. In normotensive animal studies, stimulation of peripheral OPR with a synthetic mu opioid agonist resulted in a prolonged decrease in arterial pressure [26]. Overall, the increased parasympathetic nervous system (PNS) effect and opposition to SNS resulted in a decrease in heart rate and mean arterial pressure (MAP). In addition, activation of central OPR can determine the timing of hypotension in experimental acute hemorrhage induced hypovolemia [27].

While the findings on cardiac endorphin effects in decreasing myocardial contractility and attenuating the sympathetic response are relatively new, earlier work has demonstrated similar effect with opioid anesthetics and it has been noted that renal blood flow was lower with the opioid fentanyl when compared to other anesthetics like ketamine [28]. Usually, in the range of renal auto-regulation with intact renal innervation, renal blood flow is a reflection of cardiac output. The decrease in sympathetic input from fentanyl resulted in a decrease in cardiac output and renal blood flow with compensatory rise in plasma renin.

At the level of the kidney, renal auto-regulation and blood flow along with renal oxygenation control glomerular filtration rate (GFR). With central opioid peptide receptor activation there is an increased effect of renal sympathetics, leading to vasoconstriction and renal ischemia which promote further sympathetic activity, and if persistent, can lead to ischemic AKI. This renal ischemic effect can occur with morphine, can be decreased with naloxone (a central opioid antagonist), and is shown to occur through the inhibition of renal sympathetics [29].

Among anesthetic agents, it has been noted that morphine and fentanyl decrease GFR and urine output, and this observation is among the causes for close perioperative urine output monitoring [30]. Direct and indirect effects of anesthesia and surgical stress can affect kidney function. Opioids reduce GFR and urine output. These changes are noted in the perioperative period and are considered transient as they are rapidly reversed with volume replacement [31].

Further at the level of the organ itself, it was noted that responses to intravenous morphine were dose dependent and had regional (changes in resistance in renal and mesenteric vasculature) variations in blood flow even without a major sustained change in cardiac output, blood pressure, heart rate or total peripheral resistance. While morphine may have mild effects on systemic hemodynamics, these may be more significant to local organ perfusion [32].

Questions about how opioids enhance the PNS are still being explored. The possibility of common receptors seems unlikely; a greater possibility of common second messenger pathways is still being researched. There is developing data about specific targets for opioids in the intra-cardiac ganglia expressed as mRNA and translated into specific receptor proteins in the cardiac autonomic nervous system as potential binding sites for opioids [33].

### 5.2. Opioid and the Renin–Angiotensin–Aldosterone System (RAAS) and Anti-Diuretic Hormone (ADH)

The effect of opioids on the RAAS is unclear; this may be due to RAAS changes happening as a response to alteration in MAP rather than a direct effect to the opioid itself. An exercise study in healthy normal men noted that opioids indirectly inhibited RAAS via changes in catecholamines [34]. In another study, biphalin (a synthetic encephalin opioid) and morphine both decreased MAP and blood pressure in normal and hypertensive animals; however, in experimentally-created hypertension with high angiotensin states, no decrease in MAP was noted with either biphalin or morphine [35].

It is noted that opioids increase thirst, although, the exact effect of opioids on anti-diuretic hormone (ADH) levels is unclear [36]. It could be explained as follows: pain results in non-osmotic anti-diuretic hormone (ADH) release, which is commonly called syndrome of inappropriate antidiuretic hormone (SIADH). Controlling pain with opioids would result in a decrease in ADH and a water diuresis, which then would increase thirst. The common dipsogenic effect of morphine needs an intact RAAS, and when morphine is discontinued, water retention is noted as a result of an increase in ADH along with an increase in renin and aldosterone levels [37,38].

### 5.3. Dehydration

Opioids use can lead to a decrease in fluid intake, leading to the need for close monitoring to prevent dehydration. Dehydration leading to pre-renal AKI has been traditionally considered a completely reversible condition; however, the discovery of repeated dehydration in healthy agricultural laborers in hot climates leading to a progressive form of kidney disease, called Mesoamerican nephropathy, has challenged this long held concept [39]. The mechanism postulated that the setting of repeated dehydration followed by rehydration involves proximal tubular damage. Dehydration activation of the aldose reductase pathway results in the production of fructose and oxidant damage to tubular cells [40]. This cycle is repeated with recurrent dehydration while workers toil in extremely hot temperatures and are rehydrated at inadequate intervals. This mechanism described the permanent pathology that can occur unless dehydration is treated in a timely manner.

### 5.4. Rhabdomyolysis and AKI with Opioids

In the setting of non-medical opioid use with dehydration, immobility, and with decreased respiratory drive, there is a drop in MAP, renal blood flow, GFR, and hypoxia. In addition, frequently there may be other toxins involved as with polysubstance abuse.

Myocyte hypoxia and immobility result in muscle damage, starting with hypoxia and resultant ATP depletion leading to an increase in unregulated intracellular calcium and a cascade of destruction with further muscle damage, which results in lysosomal digestion of muscle. The muscle breakdown products and other intra-cellular components are released into the serum and these include myoglobin, phosphate, potassium and other markers like creatinine phosphokinase (CPK-MM).

Myoglobin is a 17.8-kiloDalton muscle protein that is freely filtered in the kidney, endocytosed by renal tubular cells and only found in urine when present in levels above the reabsorptive capacity of the kidney. Vasoconstriction in the presence of hypoxia-induced damage to renal tubular cells furthers tubular damage and promotes acidosis. Acidosis allows for the precipitation of TammHorsfall proteins in the tubules as casts causing tubular obstruction. Now obstructed by tubular casts, further damage occurs, and this cascade of tubular damage advances upstream to decrease GFR. The ensuing hyperuricemia causes uric acid nephropathy; hyperphoshatemia leads to effects of extravascular phosphate deposition, while the hyperkalemia leads to cardiac, neuronal and muscle cell membrane instability.

### 5.5. Opioid-Related Urinary Retention

Opioids can result in urinary retention via multiple interruptions in the micturition reflex, which are thought to be partly due to its anti-cholinergic properties. Urinary retention occurs when there is impaired emptying of the bladder with a significant amount of residual urine still present in the bladder after micturition. About 10% of urinary retention is considered to be related to medication use, including those like anticholinergic respiratory inhalants, antidepressants, antipsychotics, opioids, alpha agonist and calcium channel blockers. The elderly, especially those with enlarged prostates, are at risk. With opioid use, urinary retention occurs due to the anticholinergic effect of opioids [41]. In animal studies, mu-receptor and to a lesser extent, delta receptors are noted to play a role in the centrally mediated inhibition of urinary bladder motility by opioids [42]. Mu-receptors are located in the dorsal horn of the spinal cord in the area of where afferent nerves from the bladder enter the spinal cord. When an opioid is used it could therefore impair the sensory input at the level of the dorsal horn [43]. Once these receptors are activated by the opioid it can result in the inhibition of the micturition reflex [44].

Human urodynamic studies of various opioids revealed that this finding is specific to mu receptors as non-mu opioid agonists like nalbuphine (μ-antagonist, κ-agonist) did not result in alterations in urodynamics. Once this sensory input is blocked from the bladder, there is long-lasting blockade of the volume-evoked micturition reflex that results in decreasing the usual sensation of bladder fullness. Once micturition is inhibited, greater intra-vesicular pressure (overflow pressure) by 56% was needed to induce overflow incontinence. In the period of overflow incontinence, the normal periodic vesicular contractions were lost. The increased vesicular pressure was not associated with increased urine amounts. In addition, opioids can increase bladder sphincter tone due to excessive sympathetic stimulation resulting in a bladder outlet restraint. Morphine can also bind directly to spinal receptors and result in an inappropriate complete bladder relaxation, especially of the detrusor muscle. In men undergoing renal stone extraction, pre-treatment with opioids and altered bladder sensations increased the residual volume after voiding [45]. Detrusor contraction decreased after the administration of fentanyl, while some men were noted to be unable to micturate after receiving morphine, fentanyl, or buprenorphine.

## 6. Clinical Events and Treatment of AKI Due to Opioids

Clinically, opioids can result in AKI from changes in GFR, dehydration, rhabdomyolysis and urinary retention. The presentation of opioid overdose may be with hypopnea or apnea, miosis, and stupor. The combination of decreased respiratory drive, hypoxia, a drop in renal blood flow and GFR (see mechanism above) results in renal tubular damage. Dehydration with signs of volume depletion and hypotension may be noted. Confusion or a change of mental status could be seen.

The drop in renal blood flow would activate renal sympathetics, furthering the effect of renal ischemia and tubular damage. Overall cardiac output and mean arterial pressure decreases are noted. Physical examination should include a careful evaluation for muscle tenderness; the only finding in a comatose patient may be muscle edema that could reflect the start of muscle necrosis. Abdominal examination may reveal a palpable distended bladder from urinary retention.

Serum chemistry may reveal markers of myocyte damage and expulsion of usual intracellular components into the serum like potassium, uric acid and phosphorus. Of most concern is the hyperkalemia which needs to be immediately addressed. In addition to myoglobin breakdown products, creatinine phosphokinase (CPK) may be elevated. The level of serum CPK has been correlated to the risk of acute kidney injury. In the setting of intensive care patients, a serum CPK level above 5000 U/L has been found to be twice as likely to result in AKI as compared to normal CK levels [46]. A low fractional excretion of sodium due to significant vasoconstriction, dark urine due to myoglobinuria, and tubular obstruction resulting in acute tubular necrosis with a rapid rise in creatinine and ultimately oliguria may be noted. Urinalysis reveals dipstick positive for blood (myoglobin) and urine microscopy without red blood cells as there is no bleeding.

Opioids undergo first-order elimination pharmacokinetics, whereby a constant fraction of the drug is converted to its metabolite per unit of time. However, in the case of overdose, the amount of the drug exceeds the amount removed by enzymatic conversion to its metabolite. At this level of enzyme saturation, drug kinetics shift from first order to zero order where a small increment of drug results in an unequally large rise in plasma levels and where a fixed amount, rather than a fixed ratio, of the drug can be further eliminated. These changes result in the toxicity that may be erratic, severe and delayed [47].

Once this stage is reached, with decreased kidney function, metabolism of opioids may further change depending on the specific drug. With morphine, for example, there is an increase in the mean peak concentration and the area under the concentration-time curve for both active and principle metabolites which could further worsen respiratory depression.

In the setting of methodone use, an electrocardiogram should be done early to determine if there is QT interval prolongation that could lead to arrhythmias.

## 7. Treatment

With any suspected opioid overdose where respiratory depression is found, treatment needs to be begun with antagonists like naloxone hydrochloride that can cross the blood brain barrier to reverse respiratory depression. Attention should be paid to differences in the half-life of the opioid and the antagonist used. Reversal of symptoms may be temporary as the short acting antagonist evacuates the receptor and frees it for the opioid to re-bind to it. Multiple doses of the antagonist may be needed. The half-life of naloxone is shorter than most opioids and frequent retreatment may be needed.

While it is clear that early hydration is essential to stop the vasoconstriction and allow adequate tubular flow, the quantity of fluid to be used is not clear. Intravenous hydration needs to be balanced with caution to avoid pulmonary edema, especially in the setting of pre-existing hypoxia. The ideal choice of fluid to be used is unclear as well; alkalization data only shows benefit in animal studies and there is no convincing human data to use intravenous bicarbonate. Although it is a sound idea based on pathophysiology that alkaline urine could decrease Tamm–Horsfall protein precipitation, lower the reduction reaction of myoglobin and minimize the vasoconstrictive effect of the myoglobin metabolites, no difference was noted in a large trauma study of rhabdomyolysis between hydration with 0.9% sodium chloride intravenous solution compared to intravenous bicarbonate with mannitol [46].

Once AKI occurs, if blood pressure is stable, hemodialysis needs to be considered early. Critically ill and unstable patients with AKI and rhabdomyolysis can be successfully treated by removing the excessive serum free myoglobin, with continuous venovenous hemofiltration (CVVH) for a period of 48 h. The clearance of circulating myoglobin is essential to stop further damage in patients with rhabdomyolysis while concurrently treating the cause of the rhabdomyolysis. CVVH was shown to be effective and safe in clearing myoglobin [48]. Larger molecules are better removed with ultrafiltration rather than diffusion when using continuous renal replacement therapies [49].

In patients who are suspected to have opioid overdose or those in the perioperative period who had opioid anesthesia and are unable to complain of symptoms, urine output needs to be closely monitored to determine if urinary retention is present. Bladder catheterization may be needed early in the treatment of opioid overdose if urinary retention is suspected. Bladder catheterization is essential until the effects of the opioid resolves.

If reversal of respiratory depression is initiated with an opioid antagonist, it will also result in the reversal of urinary retention. The use of naloxone may shorten the effect of urinary retention, although its effect to reverse pain control may limit the extent of use and is not routine.

The half-life of the opioid used was demonstrated to affect the risk of urinary retention in patients undergoing elective surgery. Long-acting meperidine was shown to be an independent risk factor for urinary difficulties after elective cholecystectomy [50]. The incidence of urinary retention when compared among the various modes of administration of opioids was noted to be the highest with epidural administration of opioids when compared to intravenous or intramuscular routes [51,52].

In the setting of peri-operative urinary retention, there was no consensus on what the best catheterization strategy is—either having an in-out catheterization or placing an indwelling catheter for 24 h after surgery, as there was no difference in the incidence of related urinary tract infections. The incidence of urinary retention was noted to increase with age, anorectal procedures and the use of spinal anesthesia [52]. To prevent infections with indwelling bladder catheters, they should be rapidly removed, usually within a 48 h period [53].

Another option in the peri-operative period in patients at high risk for urinary retention is to combine morphine with naloxone (an opioid antagonist) which resulted in less urinary retention without significant changes in pain control [54]. Since the use of mu receptor agonists are considered to be causative in urinary retention with the use of opioids, another option is switching to nalbuphine, which results in decreased symptoms of urinary retention while allowing for pain control and seems to be a better option than the use of naloxone [55].

## 8. Other Clinical Findings with Opioid-Induced AKI

In the gastrointestinal tract, opioids reduce propulsive peristaltic contractions, and increase muscle tone and intraluminal pressure by decreasing the release of acetylcholine in the ileum [56,57]. In the setting of acute opioid intoxication and AKI, potassium exchange resins as a treatment for hyperkalemia that work in the gastrointestinal tract have limited effectiveness and need to be used with caution (See Table 3 for opioid class effect on kidney).

## 9. Chronic Kidney Disease and opioids

### 9.1. Opioids Use Leading to CKD

Opioid use may result in CKD due to repeated episodes of AKI, toxin exposure, or infection. Toxin exposure as noted in heroin-associated nephropathy (HAN) and chronic infections as in secondary amyloidosis from skin popping, or result in a chronic infection due to the mode of drug use that then results in CKD due to hepatitis or HIV.

Frequently, multiple drugs or toxins may be used and clinical presentation may include multi-organ pathology, especially in the setting of a long-acting opioid. Autopsy data comparing methodone to heroin toxicity deaths (1000 vs. 193) revealed more pre-existing pathology with methodone including cardiac (58.9% vs. 34.5%, respectively), pulmonary (53.6% vs. 30.9%, respectively), hepatic (80.7% vs. 62.8%, respectively), and renal disease (25.0% vs. 9.5%, respectively). Further, methodone toxicity deaths included more polypharmacy with bendiazepine use (63.7% vs. 32.2%), but with less alcohol use (23.6% vs. 42.7%) [58].

### 9.2. Heroin Nephropathy: Focal and Segmental Glomerulosclerosis (FSGS), Apol1 and HIV

Heroin-associated nephropathy (HAN), which was thought to be a complication of intravenous heroin abuse, was initially recognized in the early 1970s. However, a subsequent decrease in incidence occurred toward the end of the 1989 despite an unchanged number of kidney failure incidents from other diseases like diabetes [59,60,61,62]. Analyses of surveillance records of the New York State Office of Alcoholism and Substance Abuse Services, along with reports from the New York City Department of Health, and the USA Department of Justice Drug Enforcement Administration, determined the pattern of change in use and purity of “street” heroin, and concluded that the contaminants used for chemical processing of the street heroin resulted in HAN [63].

In some cases, scopolamine was present in heroin and drug users admitted to emergency rooms with severe anticholinergic toxicity. Ninety percent of the 370 cases required admission to the hospital and half were admitted to a critical care unit [64].

Numerous studies have found clinical and pathological features of renal diseases associated with chronic parenteral abuse of heroin, morphine, and other narcotic and hallucinogenic drugs, some of which have been attributed to several adulterants. In the last four decades, illegal drug use and concurrent socio-economic burden and comorbidities including HIV and hepatitis have resulted in end stage renal disease (ESRD). It is difficult to dissect out the nephrotoxicity of each of the various components to determine the burden caused by each [65].

The renal pathology in HAN was focal and segmental glomerulosclerosis (FSGS), and it led to ESRD in a predominantly American population. HIV-associated nephropathy (HIVAN) in the African American population, which rapidly progressed to ESRD, also found FSGS on pathology [66]. Recently, *APOL1* gene variants were identified that would increase the risk of FSGS 4-fold in non-diabetic African Americans compared to Caucasian Americans, and the finding of *APOL1* gene in HIV increased the risk of HIVAN by 50% in African Americans. APOL1 expression and its effect on renal tubular podocytes and further toxins in a multiple hit theory has been suggested as a possible link between HAN and HIVAN in addition to the toxin as a cause of HAN [67,68].

### 9.3. CKD Due to Amyloidosis from Skin Popping with Opioids

In the 1980s, “skin popping” a form of intradermal injection, usually after consuming normal venous access by illicit drug users were noted to be connected to amyloidosis. Patients presented with chronic suppurative skin infections, nephrotic syndrome with peripheral edema, a bland urinary sediment and normal to large kidney on renal imaging along with renal tubular acidosis and diabetes insipidus. Rapid progression to advanced CKD was noted [69].

## 10. Multiple Classes of Pain Medication Use and CKD

A common scenario where pain control is needed is with renal calculi. In developed countries the lifetime risk of kidney stones is 8%–10% and this is higher in the elderly. Pain control of acute symptoms is accomplished with a combination of parenteral opioids and non-steroidal anti-inflammatory agents (NSAIDS) to maximize the benefits of both agents and limit doses of both to keep side effects at a minimum. The benefits of NSAIDS include a decrease of inflammation along the urinary tract. With AKI or peptic ulcer disease present, opioids may be the only option for renal colic despite being less effective with regard to inflammation [70]. In this setting it is important to monitor for opioid side effects including confusion, respiratory depression, and urinary retention [71]. While the burden of stone disease is problematic, the component of pain medications (NSAIDS vs. opioids), dehydration and AKI are difficult to dissect.

## 11. Use of Opioids in CKD

Metabolism of opioids with CKD is altered. For example, in 620 cancer patients, serum fentanyl concentrations and metabolic ratios were found to vary considerably between patients on transdermal fentanyl patches based on various factors including cytochrome genotypes and clinical factors like gender, other medications, presence of kidney disease, serum albumin and obesity [72].

In CKD, morphine has an increase in the mean peak concentration and the area under the concentration-time curve for both active and principle metabolites. With CKD, the metabolites of merperidine are present for longer and can decrease the seizure threshold and should be avoided for chronic use. Extended effects of codeine and dihydorcodeine with CKD have been reported. Pharmacokinetics of buprenorphine, alfentanil, sufentanil and remifentanil are not significantly altered in patients with renal failure [73] (see Table 4 for use of opioids with CKD).

## 12. Changes with Elderly

Prevalence of pain in the elderly is high, up to 80% in nursing home residents. Of these, 25% of them have not had any treatment for this pain [74]. Most opioids with the exception of the long-acting agents are thought to have common pharmacokinetics. Oral agents are absorbed in the gut, followed by first-pass metabolism and then are conjugated in the liver. Metabolites have varying distribution based on protein binding and are finally excreted via gastrointestinal (GI) or renal pathways. In the elderly, variations occur at multiple levels; absorption in the gut due to changes in transit time, alteration in gastric pH with concurrent use of antacids, and changes in distribution of the drug with higher adipose tissue with aging. This would increase the volume of distribution of a lipophilic drug, resulting in a longer elimination time. Decreases in hepatic blood flow due to aging or concurrent disease can also change the metabolism of the drug. In the elderly, first-pass metabolism has been noted to be altered. At the elimination stage, age-related decrease in GFR and renal blood flow can reduce elimination of drugs [75,76].

Various studies have demonstrated a decrease in renal function with aging [77]. The average use of opioids in the elderly is noted to be higher than in the overall population, and the dose of opioids needs to be adjusted for age [78]. The overlap of elderly with kidney disease makes it essential to ensure the right dose of the medication for treatment, as the usual doses may be higher than needed. For example, the pharmacokinetics of tramadol (which is excreted by the kidney) were studied after a single oral dose of in various populations including healthy controls, elderly with early CKD. Population analysis identified age as a covariate of volume of distribution (young 305 L; elderly 426 L), with a 50% longer mean elimination half-life in the elderly group without any differences in absorption processes [79,80].

Overall, due to changes with aging, suggestions about opioid use in the elderly is to start at a lower dose about 25%–50% lower than that in younger patients. In the elderly, it is suggested to avoid meperidine, propoxyphene and tramadol [41]. Meperidine metabolites could lower seizure threshold in kidney disease and should be avoided. Tramadol should be avoided in those taking serotonergic medications. Both meperidine and tramadol should be avoided in those with seizures. Codeine activation is poor in up to 30% of the population due to poor hydroxylation of debrisoquine [81].

## 13. Safe Use of Opioids in Those with Chronic Kidney Disease

Uncontrolled chronic pain is common in dialysis patients secondary to various causes like calciphylaxis, vascular steal syndromes, complications from calculi or cystic kidneys and secondary hyperparathyroidism with bone and muscle pain. In one study, 20% of patients had multi-factorial pain and more than half categorize pain as being severe despite a large portion being on opioids for pain control. About 18% had multiple causes for pain, more than half rated their pain as severe and 75% reported inadequate control of pain [82].

A systematic review of 15 studies in 12 countries among dialysis patients found variable use of opioids based on center specific treatment patterns. Prevalence of use ranged from 5%–36% and correlated positively with dialysis vintage. Pain control was reported between 17% and 38% [83]. While polypharmacy and drug interaction with negative side effects are a concern, this needs to be balanced with prevention of pain.

Opioid use in patients with renal insufficiency (excluding dialysis patients) and cancer was investigated for efficacy and tolerability in a systemic study as part of the European Palliative Care Research Collaborative’s opioid guidelines project. This systemic review of fifteen articles found that there was limited evidence of best treatment options in those with cancer and renal failure, although the authors noted that with caveats the least likely to cause harm were fentanyl, methadone and alfentanil; morphine may be associated with toxicity in renal insufficiency and increasing dosing intervals were suggested to decrease its side effects [84]. It should be noted this was for chronic cancer pain and not perioperative or acute pain control.

One group that has CKD and is treated with opioids includes those with advanced CKD who are not candidates for renal replacement therapies. They are treated with medical therapies or palliative care. Over the past decade a large number of studies have explored outcomes in those elderly either in nursing homes or with significant morbidity, and an option that the nephrology community considers beside usual care is conservative management [85]. In this group, following the World Health Organization pain control ladder, the first step would be medications like acetaminophen; a further step up would be tramadol where a lower dose and increased dosing intervals are needed with caution (see Table 3). If further pain control is needed, medications like fentanyl, alfentanil, and methodone may be needed [86]. Limited information is present for buprenorphine although it may be a reasonable choice. Hydromorphone and oxycodone have extremely limited evidence for safe use, but may be better options than morphine where there is an accumulation of metabolites.

## 14. Conclusions

With increasing global opioid use, practitioners need to be aware of the interactions between opioids and the kidney to minimize morbidity while avoiding undertreating pain. Distinctions between acute pain management and chronic pain control in those with kidney disease and the non-renal clearance of drugs in those with CKD need to be considered when controlling pain.

## Figures and Tables

**Table 1:** Classes of common opioids and relative strength compared to oral morphine 10 mg.

Type of Opioid	Dose in Milligrams
I. Exogenous	-
A. Natural	-
Morphine	10
Morphine esters	-
Morphine diacetate also called heroin	2.5
B. Semi-synthetic	-
Hydromorphone	7.5
Hydrocodone	10
Oxycodone	6.5
Oxymorphone	10
Buprenorphine (partial agonist)	0.4
C. Synthetic	-
Fentanyl (Intramuscular/Intravenous)	0.1
Meperidine (pethidine)	300
Levorphanol	1.25
Methodone	2.5
Dextropropoxyphene	130
Tramadol (also blocks reuptake of monoamines)	200
II. Endogenous	-
About 20 identified: includes endorphins, enkephalins, dynorphins, endomorphins	-

Opioids can be classified as natural or synthetic, exogenous or endogenous and are based on receptor binding ability. (See Table 2 for categories opioid receptor binders).

**Table 2:** Categories of opioid receptor binders.

**Pure Agonists**
Have no ceiling effect for analgesia.
Example: morphine and fentanyl
**Agonist-Antagonists**
Partial agonists are those drugs that have with lower efficacy to bind to the mu receptor.
Mixed agonist-antagonists are those drugs that have agonist effects at one opioid receptor and antagonist effects at another.
These are made to be less addictive.
Drugs in this category have a ceiling effect for analgesia.
Example: buprenorphine
**Pure Antagonists**
Compete for the mu receptor and block it; they are used to reverse opioid effects especially in the setting of respiratory depression. This effect may not last long and repeat treatments may be needed to keep opioids from competing andbinding to the vacant receptor.
Example: naloxone

**Table 3:** Class effects of opioids.

Anti-Diuretic Hormone (ADH)	Opioids Decrease ADH Effect
Diuretics	When added to diuretics, opioids lead to increased diuretic effect/toxicity.
Anti-cholinergics	In general opioids present a 2%–20% risk of urinary retention, with increased risk if benign prostatic hypertrophy is present. Risk is further increased if anti-cholinergics are used.
β-blockers	If combined with β-blockers, bradycardia can be enhanced.
Blood Pressure	Enhance parasympathetic effect and oppose noradrenergic effect: Opioids can lead to a clinical decrease in blood pressure.
Non-dihydropyridine Calcium channel blockers	If combined with non-dihydropyridine calcium channel blockers, bradycardia can be enhanced.
Constipation	Due to decreased acetylcholine release in the gut from opioids.
Central nervous system (CNS) depressants (alcohol/benzodiazepenes etc.)	Opioids interact with other CNS depressants for additive effect.
Desmopressin	Opioids can enhance the effect of desmopressin.
Magnesium sulphate	With magnesium sulphate CNS depression can be increased.
Sodium polystyrene sulfonate	Use with caution in the setting of hyperkalemia with renal failure; (with constipation caution is needed regarding use of potassium resin exchangers in the gut).

**Table 4:** Opioids and use in chronic kidney disease (CKD). In general, with CKD use opioids with caution; with decreased renal function, dose and timing interval may need to be adjusted. This is particularly true for agents that are used for chronic pain where a steady state has been reached with distribution of drug and metabolites into various compartments (versus limited use peri-operatively or for anesthesia). While opioids are used safely in CKD for both pain control and anesthesia, it is the chronic use of opioids and the level of CKD that alter the pharmacokinetics of the drugs and causes metabolites to accumulate that lead to unwanted side effects. With CKD, it is important to note that the non-renal clearance of medications is altered with variations in the phases of drug metabolism. In addition, it is with chronic use that accumulation and steady-state distribution of drugs, intermediate compounds and metabolites in various compartment occur, which may result in unwanted side effects. HD: hemodialysis; Vd: volume of distribution. Molecular weight (MW) in daltons; in general, small molecules that are less than 500 daltons, with a small Vd and are not protein bound may be removed easily with HD.

Drug	Use in End Stage Renal Disease (ESRD) and Chronic Kidney Disease
Morphine	ESRD: Use with caution and start with small doses as metabolites accumulate and may have delayed respiratory depression; pruritus may be difficult to control. Up to 47% can be removed with HD despite being protein bound (MW 668 dalton; 35% protein bound, Vd = 1–6 L/kg).
	CKD: Starting oral dose is 25%–50%; start low dose and titrate slowly with longer dosing intervals as metabolites may accumulate with a delayed effect of respiratory depression.
	Drug interactions: nimodipine augments morphine’s analgesic effect via unknown mechanisms. May need small doses of morphine when using nimodipine.
Diamorphine (Heroin/morpine diacetate)	Not medically used in USA as quickly addictive.
	ESRD: Use with caution due to accumulation of metabolites (MW 369; not protein bound; easily crosses into brain; pro-drug converted to morphine and metabolites).
	CKD: Use with caution due to accumulation of metabolites.
Codeine	ESRD: Significant accumulation of metabolites ; start at 50% of dose with increased interval of dosing and carefully titrate (MW 299; Vd = 2.6 L/kg; 90% excreted by kidneys).
	CKD: GFR < 50 mL/min: start at 75% dose with increased interval and carefully titrate due to accumulation of metabolites.
Tramadol	ESRD: As it is dialyzed out administer on day of HD; increased interval of dosing to 12 h may be needed (MW 263; Vd 2.6–2.9 L/kg; 20% protein bound).
	CKD: For GFR < 30 mL/min, increase dosing interval to 12 h; avoid the use of extended release; for GFR > 30 m/min, use with caution.
	Can have symptoms of urinary frequency and urinary tract infection; metabolized in liver, metabolites excreted via kidney.
Oxymorphone	ESRD: No clear data of use (MW 301; Vd 3 L/kg).
	CKD: eGFR > 50 mL/min reduce starting dose 50% and increase dosing frequency for titration; bioavailability increases with CKD.
	Thromobtic thrombocytopenic purpura and acute kidney injury (AKI) noted when the tablets are crushed and intravenously administered as in injection with drug abusers.
Hydromorphone	ESRD: Limited information with fluctuating levels may be difficult to use due to removal of drug; clearance with HD 105 mL/min with change in drug level of 55% with one treatment of HD; parent drug is removed but metabolites may persist and accumulate; (MW 285; Vd 1.2 L/kg; 8%–19% protein bound).
Oxycodone	ESRD: Use with caution; limited information case reports (MW 315; Vd 2.6 L/kg; 45% protein bound).
	CKD: Serum concentration increases 50% if GFR < 60 mL/min.
	Limited information suggests starting at one-third the usual starting dose and increasing interval between dosing as half life increases.
	Drug interaction: Conivaptan: could increase concentration of CYP3A4 substrate.
Buprenorphine	ESRD: May be used with caution, limited information (MW 467; Vd 188–335 L/kg; 96% protein bound). Transdermal route may be satisfactory.
	CKD: May be used with CKD.
Methodone	ESRD: Dose at 75% normal dose; lipophilic and minimal clearance with HD with less than 15% change in levels); methodone clearance with HD is about 17–19 mL/min (MW 345; 85%–90% protein bound; Vd 1–8 L/kg).
	CKD: eGFR > 10 mL/min no change in dose needed.
	If QTc prolonged on EKG, an alternate drug may be needed.
	Drug interaction: Conivaptan: could increase concentration ofCYP3A4 substrate.
Fentanyl	ESRD: Transdermal patch suggested to not be used with ESRD (MW 336; lipophilic; Vd 4 L/kg).
	CKD: Reduce dose by 50%; limited information on other forms besides transdermal route; lipophilic and protein bound; free fraction increases with acidosis.
	Conivaptan: Could increase concentration of the CYP3A4 substrate.
